# *Astragalus* Polysaccharide RAP Selectively Attenuates Paclitaxel-Induced Cytotoxicity Toward RAW 264.7 Cells by Reversing Cell Cycle Arrest and Apoptosis

**DOI:** 10.3389/fphar.2018.01580

**Published:** 2019-02-11

**Authors:** Wan-Rong Bao, Zhi-Peng Li, Quan-Wei Zhang, Li-Feng Li, Hong-Bing Liu, Dik-Lung Ma, Chung-Hang Leung, Ai-Ping Lu, Zhao-Xiang Bian, Quan-Bin Han

**Affiliations:** ^1^School of Chinese Medicine, Hong Kong Baptist University, Kowloon Tong, Hong Kong; ^2^Department of Chemistry, Hong Kong Baptist University, Kowloon Tong, Hong Kong; ^3^State Key Laboratory of Quality Research in Chinese Medicine, Institute of Chinese Medical Sciences, University of Macau, Macau, China

**Keywords:** *Astragalus* polysaccharide, cytotoxicity, protective effect, cell cycle, apoptosis

## Abstract

**Purpose:** The purpose of this study was to determine if an *Astragalus* polysaccharide (RAP) can protect immune cells from the toxic side effects of paclitaxel (Taxol), a powerful anti-tumor drug whose equally powerful side effects limit its clinical use.

**Methods:** We hypothesized that RAP can reduce the toxic effects induced by Taxol. To test this hypothesis, we conducted a series of studies *in vivo* and *in vitro*. First, we confirmed RAP’s effects *in vivo* utilizing BALB/c mice inoculated with 4T1 mouse breast cancer cells as the tumor model. Mice were treated with RAP and/or Taxol, and the differences in the life spans were recorded. Second, a co-culture cell model was used to study the protective effect of RAP on cells vis-a-vis Taxol. The cell cycle and apoptosis of RAW 264.7 cells that were treated with RAP with/without Taxol were checked by flow cytometry and Hoechst staining. Proteins involved in the cell cycle and apoptosis were also tested by Western blot to reveal the probable mechanism.

**Results:** RAP prolonged the life span of tumor-bearing mice treated with Taxol. The *in vitro* experiments showed that Taxol suppressed the proliferation of RAW 264.7 cells while RAP protected the RAW 264.7 cells from Taxol-induced suppression. The protection is selective because RAP had no effect on 4T1 cells. Furthermore, Taxol clearly led to cell cycle arrest mainly at the G2/M phase and generated cytotoxicity against RAW 264.7 cells, while RAP blocked cell cycle arrest and protected cells from apoptosis. Taxol up-regulated the protein levels of P-H_2_A, PARP, Chk1, p53, and p21 and down-regulated Bcl-Xl and Mcl-1, and RAP reversed the expression of all these proteins.

**Conclusion:** These results suggested that RAP can protect immune cells from Taxol-induced toxicity, by changing the cell cycle and apoptosis.

## Introduction

Paclitaxel (Taxol), a classic microtubule-targeting agent, is one of the most useful antineoplastic agents (Pellegrini and Budman, 2005; Wani and Horwitz, 2014; Weaver, 2014). It binds to tubulin (Yang et al., 2016). This binding results in a cascade of disruptions ultimately ending in cancer cell death. First, this binding changes the dynamic equilibrium between assembly and disassembly of microtubules, which actively prolongs mitotic arrest (Yang and Horwitz, 2017). It also disrupts the cytoskeletal framework that is necessary for tumor cell replication and metastatic spread (Magidson et al., 2016; Zhang et al., 2018), and this disruption subsequently triggers cancer cell death not only in mitotic arrest state, but also after mitotic slippage to an abnormal G1 (Zhu et al., 2014). Taxol has been commonly prescribed to treat a variety of tumors, particularly ovarian and breast cancer (Reichman et al., 1993; Kampan et al., 2015; Notte et al., 2015; Bo et al., 2016; Liu et al., 2016).

In addition to its advantages, Taxol also, unfortunately, induces some cytotoxic effects, such as neurotoxicity, hypersensitivity reactions, hematologic toxicity, cardiac disturbances, and gastrointestinal tract symptoms. These side effects have severely limited its optimal clinical application as an anti-cancer agent (Kober et al., 2017). Some compounds have been reported to reduce its cytotoxicity (Visconti and Grieco, 2017). For example, *Vernonia amygdalina* (Bitter Leaf Plant; Asteraceae) has been reported to improve the anticancer effects of Taxol against breast cancer, while reducing harmful side effects (Howard, 2016). Mito VitE was reported to have the ability to abrogate the mitochondrial function and glutathione in DRG cells affected by Taxol, without decreasing cancer cell cytotoxicity (McCormick et al., 2016). Fibrates can also be used to reduce the vascular endothelial dysfunction induced by Taxol (Watanabe et al., 2015). Other reagents and methods such as those involving nanoparticles, bevacizumab (Miller et al., 2007) and doxorubicin (Sikov et al., 2015) have also been tested with Taxol to reduce its cytotoxicity or improve its anticancer effect (Ruttala and Ko, 2015). Unfortunately, most of agents themselves are also chemotherapeutic and have some safety concerns, e.g., cardiac toxicity and neutropenia (Razis and Fountzilas, 2001; Yoneyama et al., 2017). Furthermore, the underlying mechanism has not been studied extensively.

Chinese medicines in combination with paclitaxel was reported to significantly decrease the risk in 729 patients with advanced breast cancer in the clinic (Lee et al., 2014). In another clinical trial, which used 314 patients to evaluate the effect of Traditional Chinese Medicine (TCM) as a combination medication with adjuvant chemotherapy, Radix Astragali was used to strengthen the healthy qi and eliminate pathogenic factors for patients. The skeleton component of the Chinese Medicine formula used in this clinical trial is Radix Astragali which is often used as an edible tonic herb for improving the immune system and strengthening the physique (Jiao et al., 2017). Polysaccharides are believed to be the major active ingredients in Radix Astragali (Song et al., 2008), and have demonstrated its immune-modulatory, anti-tumor (Jung et al., 2016), anti-virus (Chen et al., 2015), and inflammatory properties (Auyeung et al., 2016). RAP, a major polysaccharide purified from Radix Astragali in our previous work, has been studied in terms of its immune-modulatory and anti-tumor properties. Our results showed that RAP affected the cytokine profile of unstimulated human peripheral blood mononuclear cell (PBMC). RAP was able to stimulate the expression of IL-1 and TNF-α, which are important in bacterial immune responses. It can also induce the expression of IL-10, IL-12, and GM-CSF. The fact that these cytokines are related to monocytes suggested that RAP is an activator of monocytes (Yin et al., 2012). Further studies have revealed that TLR 4 is a receptor of RAP and can mediate its immune activities, since RAP activated TLR 4 related MAPKs, including phosphorylated ERK, phosphorylated JNK, and phosphorylated p38, and induced translocation of NF-κB as well as degradation of IκB (Wei et al., 2016).

Our preliminary study found that RAP preserved RAW 264.7 cells in normal cell activity and cell profile from the impact of Taxol within 72 h using a live cell image system (data not shown). The current study was used to confirm the protective effect of RAP to Taxol and explore the underlying mechanism in terms of cell cycle arrest and apoptosis.

## Materials and Methods

### Reagents

Paclitaxel (Taxol) was purchased from Sigma-Aldrich, Inc. (St. Louis, MO, United States). Phospho-Histone H_2_A. X, PARP, Bcl-Xl, Mcl-1, Chk 1, and p53 antibody were provided by Cell Signaling (Beverly, MA, United States). The antibody of p21 was purchased from Santa Cruz Biotechnology (Santa Cruz, CA, United States).

### Cell Lines and Mice

4T1 mouse breast cancer cell lines and RAW 264.7 murine macrophage cell lines were originally obtained from the American Tissue Culture Collection. Cells were grown in a 35 mm dish at 37°C in a humidified atmosphere of 5% CO_2_ and were maintained as monolayer cultures in Dulbecco’s minimal essential medium supplemented with 10% fetal bovine serum, 100 U/mL penicillin and 100 μg/mL streptomycin.

BALB/C mice, aged 6–8 weeks (18–20 g) were purchased from the Chinese University of Hong Kong and maintained under clean conditions in an animal room in Hong Kong Baptist University. The animals had free access to food and water in animal cages that were maintained in a pathogen-free environment (24 ± 1°C, humidity of 55 ± 5%) with a 12/12 light cycle.

### RAP Preparation

The roots of *Astragalus membranaceus* were purchased from a herbal store in Hong Kong and identified by Dr. Chun-Feng Qiao. The voucher specimens are deposited at the Institute of Chinese Medicine, the Chinese University of Hong Kong, with voucher specimen number 2010–3268 (Yin et al., 2012). The isolation and purification procedure was performed according to the previous study (Yin et al., 2012). Briefly, the air-dried Radix Astragali was powdered and extracted twice with boiling water. The solution was filtered, combined, and concentrated. The solution was precipitated with absolute ethanol. The precipitate was resolved again in water and deproteinated. The water solution was then dialyzed. Finally, the retentate was lyophilized. The product was dissolved in distilled water again and separated with a Hiload 26/60 Superdex-200 column that was eluted with water. Fractions were collected, dialyzed, and finally lyophilized to obtain RAP. The RAP used in this study was not contaminated with endotoxin.

RAP is a water soluble polysaccharide (Yin et al., 2012). Its structure was elucidated by monosaccharide composition, partial acid hydrolysis and methylation analysis, and further confirmed by FT-IR, GC–MS and ^1^H and ^13^C NMR spectra, SEM and AFM microscopy. Its average molecular weight was 1334 kDa and composed of Rha, Ara, Glc, Gal, and GalA in a molar ratio of 0.03:1.00:0.27:0.36:0.30. The backbone of RAP consisted of 1,2,4-linked Rhap, α-1,4-linked Glcp, α-1,4-linked GalAp6Me, β-1,3,6-linked Galp, with it branched at O-4 of the 1,2,4-linked Rhap and O-3 or O-4 of β-1,3,6-linked Galp. The side chains mainly consisted of α-T-Araf and α-1,5-linked Araf with O-3 as branching points, having trace Glc and Gal. The terminal residues were T-linked Araf, T-linked Glcp, and T-linked Galp.

### Mice Mammary Tumor Model Establishment and Treatment

All animal operations were performed according to the guidelines of the Animal Experimentation Ethics Committee of Hong Kong. The mice were housed in groups of four to five animals per cage for 1 week before the experiment. To establish the tumor model, well cultured 4T1 cells (4 × 10^5^/each mouse) were collected and washed 3 times before re-suspended in 0.2 ml phosphate buffered saline (PBS). The cells were subcutaneously (s. c.) inoculated into the mammary fat pad of each mouse. The tumor-bearing mice were randomly divided into four groups (*n* = 10): Control group (dH_2_O, oral gavage), RAP group (40 mg/kg/day/mouse, oral gavage), Taxol group (20 mg/kg/week/mouse, intraperitoneal injection) and RAP+Taxol group (RAP 40 mg/kg/day/mouse+Taxol 20 mg/kg/week/mouse). In groups receiving RAP, RAP was initiated 10 days before cancer cell implantation and was continued every day thereafter. During and after the treatment lasting for 6 weeks, deaths of mice were recorded every day, and survival rate was monitored until the end of the period.

### MTT Assay

4T1 and RAW 264.7 Cells were plated in 96-well plates at 5 × 10^4^ cells (100 μL) per well. The next day, cells were treated with a different concentration of RAP (12.5, 25, 50, 100 μg/mL) in three replicates for 24 h. Following incubation, MTT solution (5 mg/mL in PBS) was added to each well and the plates were incubated at 37°C for another 4 h. The medium was then discarded and DMSO was added to dissolve the formazan crystals. The absorbance of each sample was read at 490 nm using a Benchmark Plus microplate reader (Bio-Rad, Richmond, CA, United States).

### Cells Co-culture Model

The effect of RAP with/without Taxol on the cell viability of 4T1 cells and RAW 264.7 cells in a co-culture model was measured by MTT assay. As Figure 2A showed, 4T1 cells (2 × 10^5^) were first planted on the 24-well plates, then the RAW 264.7 cells (2 × 10^4^) were seeded on the collagen-coated polycarbonate transwell membrane (corning, 6.5 mm diameter, 3.0 μm pore size), and were put inside the wells of 24-well plates (cultured together with 4T1 cells). 4T1 and RAW 264.7 cells alone were added to 24-well plates as a control. Culture medium (0.1 mL within the insert, 0.6 mL in the outer well) was replaced after 24 h, then cells were incubated with different concentrations of RAP (12.5–100 μg/mL) with/without Taxol (10 μM) for 24 h. Following incubation, MTT assay was used to measure the cell proliferation.

### Cell Cycle and Apoptosis Analysis by Flow Cytometry

The cellular DNA content of treated RAW 264.7 cells was determined by staining cells with PI and measuring on a flow cytometer. Well cultured RAW 264.7 cells (2 × 10^6^/well) were seeded in a 6-well plate and then treated with RAP (50 μg/mL) with/without Taxol (10 μM). After 24 h, cells were collected and washed twice in PBS, followed by fixation in 70% ethanol on ice for 2 h. Cells were then re-suspended in 0.45 mL PBS and 25 μL of 2 mg/mL RNase A at 37°C for 15 min, and finally stained with 20 μL of 1 mg/mL PI for 30 min, before detection with a flow cytometer.

Apoptotic cells were labeled using an Annexin V-FITC/ PI apoptosis detection kit (Sigma, United States) and then also detected using the Flow cytometer. Briefly, cells were separated into 4 groups as before, after 24 h, cells were collected and washed twice with cold PBS and re-suspended in 500 μL buffer, then measured with a flow cytometer.

### Hoechst 33342 Staining

Replicate cultures of 2 × 10^5^ RAW 274.7 cells per well were plated in 24-well plates. RAP (50 μg/mL) with/without Taxol (10 μM) were added to cells after 24 h. After treatment for 24 h, cells were incubated with 5 μL of Hoechst 33342 solution (Sigma, United States) per well at 37°C for 10 min, followed by observation under a fluorescence microscope. Fluorescence was observed in the nuclei and multiple nuclei could be detected at higher magnifications.

### Western Blot

Replicate cultures of 1 × 10^6^ RAW 274.7 cells per well were seeded in 6-well plates, then treated with RAP (50 μg/mL) with/ without Taxol (10 μM) for 24 h. After that, cells were washed twice with phosphate buffered saline and harvested in cold lysis buffer containing protease inhibitors or phosphatase inhibitors. Cell lysates were collected from culture plates, and protein were collected by centrifugation. The concentrations of proteins were determined by a BCA protein assay (Pierce Biotechnology, Rockford, IL, United States). The total protein was boiled in 4× loading buffer (Bio-Rad, United States) for 10 min at 100°C, then loaded into various concentrations of Tris–HCl-Polyacrylamide gels according to its molecular properties (from 6 to 12%), and transferred electrophoretically to an Immobilon-P membrane (Millipore Corporation, Billerica, MA, United States). Membranes were incubated with primary antibodies and appropriate horseradish peroxidase-labeled secondary antibodies. Membranes were additionally probed with an antibody against β-actin (Cell Signaling) to normalize the loading of proteins among samples. In the last step, secondary antibodies were detected by chemiluminescent agents (Pierce Biotechnology).

### Statistical Analysis

All data were expressed as mean ± SD/SEM. Statistical analysis was performed using one way ANOVA, two way ANOVA and t tests, with *P*-values (P) < 0.05 regarded as statistically significant. Each experiment was repeated at least three times, and each data point represents the mean of at least three parallel samples. All calculations were performed using Prism 5 software.

## Results

### RAP Increased the Survival Rate in Mammary Tumor Model Mice

**FIGURE 1:**
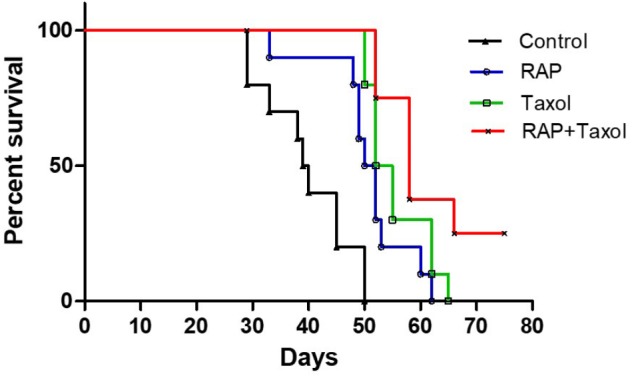
Long time survival of 4T1 breast tumor-bearing mice treated with RAP with/without Taxol.

Seven days after BALB/c mice were inoculated with 4T1 mouse breast cancer cells, the tumor model was established successfully. As shown in Figure 1, the control group of mice began to die on day 30 post tumor inoculation, whereas RAP, Taxol and RAP+Taxol treated groups did not begin to die until day 35, 50, and 52, respectively. By day 50, all of the control group mice had died. In contrast, the survival rates in the treated groups were still 50–100%. Mice in the RAP+Taxol combination group were surviving at a rate of 25% when the mice in the RAP or Taxol alone groups had all died. The data was analyzed using a Log-rank Test. Compared with control group, the RAP group, Taxol group and RAP+Taxol group showed significant differences (*p* < 0.0001). Compared with Taxol group, RAP+Taxol group demonstrated a significant difference (*p* < 0.05). As for tumor diameter and body weight, compared with Taxol group, RAP+Taxol group had no significant difference (Supplementary Figure S1). These observations indicated that RAP might not be able to affect Taxol’s efficacy to cancer cells *in vivo*, while the combined treatment of RAP and Taxol generated synergism in term of improving survival rate than either individual therapy.

### RAP Reduced the Taxol-Induced Cytotoxicity Without Affecting Its Anti-tumor Efficacy

**FIGURE 2:**
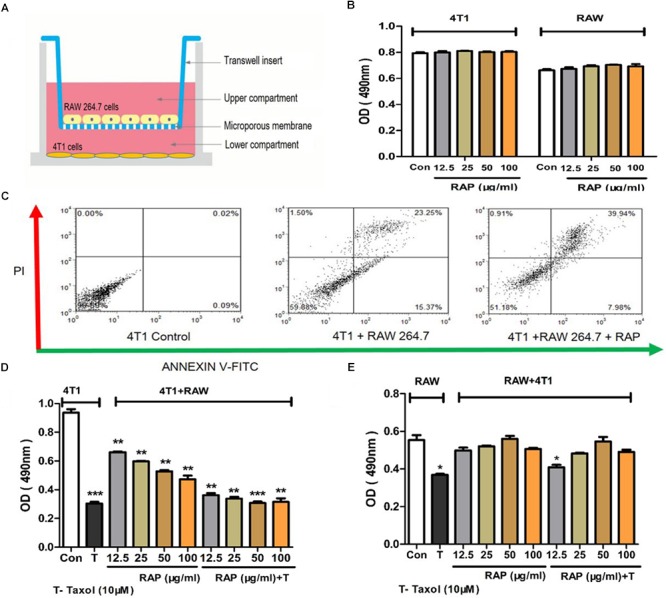
Effects of RAP with/without Taxol on 4T1 and RAW 264.7 cells. **(A)** 4T1 and RAW 264.7 cells co-culture model; **(B)** 4T1 and RAW 264.7 cells were not stimulated by RAP directly; **(C)** Apoptosis of 4T1 cells in co-culture system induced by RAP (50 μg/mL); **(D)** Proliferation of 4T1 and **(E)** RAW 264.7 cells in co-culture model treated by RAP with/ without Taxol. Data are presented as mean ± SD. Compared with the control group. ^∗^*p* < 0.05, ^∗∗^*p* < 0.01, ^∗∗∗^*p* < 0.001.

We assumed that the ability of RAP to help Taxol in fighting tumor cells were due to its capacity to protect the immune cells but not the direct toxicity effect to the tumor cell itself. In order to test this hypothesis, RAP’s effect on the proliferation of 4T1 cells and RAW 264.7 cells was detected. Because cancer cells and immune cells might affect each other under real situations, RAP’s effect on 4T1+RAW 264.7 cells was also examined in an *in vitro* co-culture model. In all three cell experiments, cells were treated with different doses of RAP from 12.5–100 μg/mL with/ without Taxol (10 μM) for 24 h *in vitro*. As shown in Figure 2B, RAP didn’t induce the proliferation of 4T1 cells or RAW 264.7 cells directly. However, in the co-culture model (Figure 2C–E), RAP suppressed the growth of 4T1 cells but exhibited no effect on RAW 264.7 cells. As we reported previously, RAP treatment could induce RAW 264.7 cell’s inhibition against 4T1 cells in a dose-dependent manner. Of interest, RAP did not disturb the Taxol’s cytotoxic effect against 4T1 cells but exhibited protection to taxol-treated RAW 264.7 cells. In other words, RAP selectively protected RAW 264.7 cells from the toxicity of Taxol without reducing Taxol’s toxic effect toward cancer cells. This conclusion was supported by the results of an apoptosis assay in co-culture model (Figure 2C).

### RAP Attenuated G2/M Cell Cycle Arrest of RAW 264.7 Cells Induced by Taxol

**FIGURE 3:**
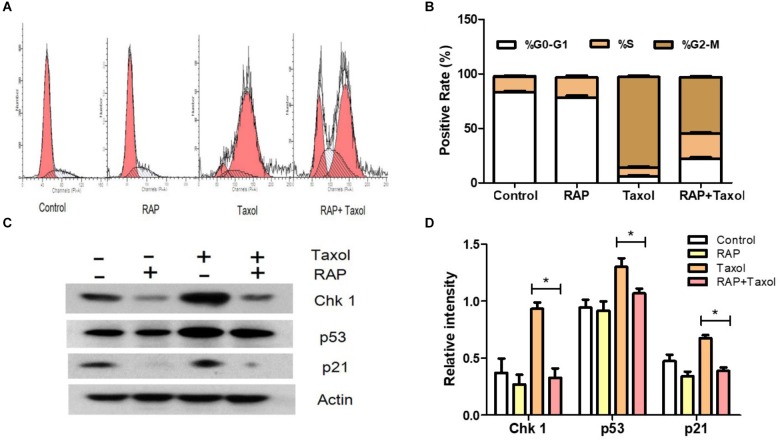
G2/M cell cycle arrest and related proteins of RAW 264.7 cells treated by RAP (50 μg/mL) with/without Taxol (10 μM). **(A)** Diagram of cell cycle analysis in RAW 264.7 cells by flow cytometry; **(B)** Statistical analysis of G0-G1, S, and G2/M populations in RAW 264.7 cells; **(C)** Western blotting analysis of the level of phosphorylation of Chk1 (MW: 56 kDa), p53 (MW: 53 kDa), and p21 (MW: 21 kDa) protein expression; **(D)** Quantitative analysis of Chk 1, p53, and p21 (^∗^*p* < 0.05 compared with Taxol alone group).

To investigate whether the cell cycle arrest of RAW 264.7 cells could be induced by RAP with/without Taxol, the cell cycle percentage of treated RAW 264.7 cells were detected primarily by flow cytometry analysis after PI staining. As shown in Figure 3A,B, after RAW 264.7 cells were treated with RAP (50 μg/mL) with/ without Taxol (10 μM) for 24 h, changes appeared in the percentage of RAW264.7 cells at the G0-G1, S, and G2/M phases. In Taxol alone and RAP+Taxol groups, the percentage of cells at the G0-G1 phases decreased from 86 to 18% and 22%, respectively. However, cells in the G2/M phase increased from 5 to 83% and 48%. For S phase, the cell percentage only changed slightly. The results suggested that Taxol arrested the cell cycle mainly at the G2/M phase. RAP alone had no significant effect on cell cycle, but it obviously disturbed the G2/M phase arrest effect of Taxol when added together with it.

Signals from the transducer to effector kinases are forwarded by mediator proteins such as Chk1, a Ser/Thr protein kinase which controls the G2/M phase transition in response to DNA damage. p53 (a tumor suppressor) activates p21 (a cyclin-dependent kinase inhibitor, which represents a major target of p53 activity), both of which have also been well established as critical mediators of cellular responses to DNA damage, apoptosis and cell cycle arrest (Becker et al., 2014; Zeng et al., 2014; Zhu et al., 2014). Therefore, we focused our attention on these checkpoint-related proteins. As illustrated in Figure 3C,D, Taxol promoted the protein expressions of Chk 1, p53 and p21, while RAP reversed these changes.

### RAP Suppressed the Apoptosis of RAW 264.7 Cells Exposed to Taxol

**FIGURE 4:**
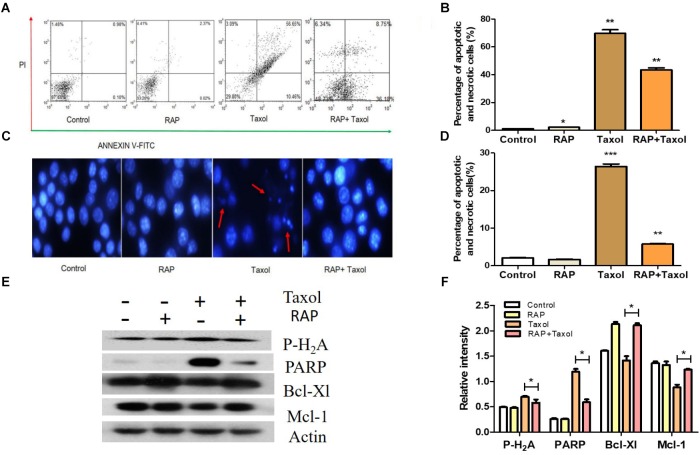
Apoptosis of RAW 264.7 cells treated by RAP (50 μg/mL) with/without Taxol (10 μM). **(A)** Apoptotic effect of RAP with/without Taxol on RAW 264.7 cells detected by flow cytometry; **(B)** Statistical analysis of apoptotic effect of RAP with/without Taxol detected by flow cytometry; **(C)** Apoptosis that assessed by Hoechst 33342 staining: red arrow showed some of the typical “apoptotic bodies.” **(D)** Statistical analysis of apoptotic effect of RAP with/without Taxol detected by Hoechst staining; **(E)** Western blotting analysis of Phospho-Histone H2A. X (MW: 15 kDa), PARP (full length, MW: 116 kDa), Bcl-Xl (MW: 30 kDa) and Mcl-1 (MW: 35 kDa); **(F)** Quantitative analysis of Phospho-Histone H2A. X, PARP, Bcl-Xl, and Mcl-1 (^∗^*p* < 0.05 compared with Taxol alone group). Data are presented as mean ± SD. Compared with the control group. ^∗^*p* < 0.05, ^∗∗^*p* < 0.01, and ^∗∗∗^*p* < 0.001.

Apoptosis in the RAW 264.7 cells treated with RAP and with/without Taxol for 24 h were measured using flow cytometry. As shown in Figure 4A, in cells treated with RAP alone, only small percentages (2.39%) of cells underwent apoptosis, while in cells treated with Taxol, the percentage of apoptotic and necrotic cells (expressed as Annexin V positive/ PI negative and Annexin V/PI positive cells) increased to 67.11%. But this effect of Taxol was blocked by RAP as demonstrated by the RAP+ Taxol group, in which the percentage of these cells fell to 44.93%. The percentage of apoptotic cells of RAW 264.7 cell which induced by RAP with/without Taxol is directly compared in Figure 4B. The protective effect of different dosages of RAP can also be detected using flow cytometry. Compared with Taxol group, RAP (10–100 μg/mL)+Taxol groups had significant protective effect to RAW 264.7 cells, while there were no significant differences between different RAP dosage groups (Supplementary Figure S2). The nuclear damages were observed by Hoechst staining in control and RAP with/ without Taxol groups. The typical condensation and fragmentation of the nuclei’s were easy to be observed in Taxol group, while disappearing in RAP combine Taxol group (Figure 4C,D). The results shown here demonstrated that Taxol is toxic to RAW 264.7 cells, but RAP can protect them from this toxicity.

It is well established that Taxol-mediated DNA damage triggers apoptosis. In order to find the related probable mechanism, Phospho-Histone H_2_A, and PARP (both are DNA damage markers) and Bcl-Xl and Mcl-1 (both are negative regulators of apoptosis) were measured (Khorasanizadeh, 2004; Bertini et al., 2011). The results from western blot analysis further confirmed this assumption. As shown in Figure 4E,F, Taxol treatment up-regulated the expression levels of Phospho-Histone H_2_A, and PARP, and down-regulated Bcl-Xl and Mcl-1. However, all these changes were significantly reversed by RAP treatment.

## Discussion

One of the main barriers in conventional anticancer therapy is its toxicity to normal tissues. Up until now, the specific clinical approaches that can be used to reduce the chemotherapy-mediated toxicities are rare. Recently, several studies have reported that herbal extracts originated from TCM could reduce chemotherapy-induced side effects (Chen et al., 2016; Liu X. et al., 2017). For instance, the PHY 906, which was based on the Huang Qin Tang, can reduce CPT-11 induced gastrointestinal toxicity, and this finding was further supported by a clinical trial (Lam et al., 2010; Liu and Cheng, 2012). TCM also can be used to improve the white cell counts in women undergoing chemotherapy for ovarian cancer (Chan et al., 2011). What is more interesting in our study is that RAP is the major polysaccharide of Radix Astragali which is an edible tonic herb that can be used for a long time without any toxicity concern. Actually, Radix Astragali has been used to improve the Qi of small lung cancer patients (Jiao et al., 2017). TA-65, a proprietary exact from Radix Astragali, was associated with a significant age-reversal effect (Liu P. et al., 2017). These studies and our results suggest that RAP has great potential in cancer therapy.

The mechanism underlying the protective effect of RAP is complicated. RAP may bind to many target molecules, then trigger subsequent signal cascades, many of which are interconnected. We used RAW 264.7 cells as a model to study the probable mechanism of the protective effect *in vitro* since the macrophage is one of the most sensitive immune cells responding to RAP and Taxol in splenocyte of mice (data not shown). In the part of cell cycle, similar to reported work (Zhu et al., 2014), we found that Taxol increased the expression of cell cycle protein Chk1, while RAP decreased it to almost normal levels. In the part of signaling pathways involved in Taxol-induced cytotoxicity, involving p38, JNK, ERK (El-Khattouti et al., 2014; Park et al., 2016), we didn’t find any relationship between those signals and RAP’s effect (data not shown). Instead, we found that activation of both p53 and p21 played a critical role in RAP’s ability to protect normal cells from Taxol-induced apoptosis. Combination with RAP decreased the high expression of p53 and p21 induced by Taxol. Recent work by Becker et al. also showed that p53 is a key factor of Roc-A, a Chinese medicine formula which showed a protective effect against chemotherapy (Becker et al., 2014).

According to previous studies, two distinct sets of biomarkers needed to be considered in chemotherapy-induced apoptosis. One set were those controlling DNA damage-dependent post-slippage death, for example p53 and its involving genes; another set were genes that regulate cell death during prolonged mitotic arrest, such as Bcl-Xl and Mcl-1 (Zhu et al., 2014). The formation of multiple small nuclei was also a marked feature of cells that slip out of Taxol-induced mitotic arrest (Shi et al., 2011; Zhu et al., 2014). In our study, RAP inhibited the apoptosis of RAW 264.7 cells affected by Taxol. Less nuclear fragmentation was founded in the RAP+Taxol group, compared with Taxol alone. RAP also successfully inhibited the Taxol-induced H_2_A and PARP expression, and reversed the expression of Bcl-xl and Mcl-1. Given that RAP can reverse the changes in cell cycle and apoptosis induced by Taxol, it is reasonable to suggest that RAP may have the function, at least in part, to protect normal cells from the cytotoxicity induced by Taxol.

Normally, polysaccharides are believed to have low oral bioavailability, due to their high molecular weight and low stability in the digestive tract (Peltier et al., 2006; Han, 2018). Actually, some polysaccharides could be digested in gut tract, but not all. Previous studies have reported that orally administered β-1,3-glucans can be transferred to spleen lymph nodes and bone marrow with the help of macrophages (Hong et al., 2004). Because we have found that RAP showed strong immune-modulating effects on macrophages, it is also possible for RAP to directly interact with the main immune organ of the small intestine, the Peyer’s patch. Another possibility has been highlighted by more and more literature addressing the importance of gut microbiota in beneficial effects of fiber diet. Like fiber, RAP possibly functions as optimum food for some specific gut bacteria, modulating the composition and function of gut microbiota which subsequently affects the health of the host body. The weakness in conventional bioavailability of RAP might not be problematic enough to weaken its clinical potential when more evidences are obtained in the future.

## Conclusion

The results obtained in this study indicated that RAP treatment effectively reduced the cytotoxicity induced by Taxol. It appears to work by reversing the changes in cell cycle and apoptosis that are caused by Taxol. The efficacy of the RAP-Taxol combination warrants further study.

## Author Contributions

W-RB and Q-BH designed the experiments and wrote the manuscript. W-RB, H-BL, and L-FL performed the experiments. Z-PL, Q-WZ, and Q-BH contributed in data analysis. D-LM, C-HL, Z-XB, and A-PL provided the reagents, materials, and analysis tools.

## Conflict of Interest Statement

The authors declare that the research was conducted in the absence of any commercial or financial relationships that could be construed as a potential conflict of interest. The handling Editor declared a shared affiliation, though no other collaboration, with several of the authors.

## References

[B1] Auyeung K. K. Han Q. B. Ko J. K. (2016). Astragalus membranaceus: a review of its protection against inflammation and gastrointestinal cancers. *Am. J. Chin. Med.* 44 1–22. 10.1142/s0192415x16500014 26916911

[B2] Becker M. S. Schmezer P. Breuer R. Haas S. F. Essers M. A. Krammer P. H. (2014). The traditional Chinese medical compound Rocaglamide protects nonmalignant primary cells from DNA damage-induced toxicity by inhibition of p53 expression. *Cell Death Dis.* 5:e1000. 10.1038/cddis.2013.528 24434508 PMC4040689

[B3] Bertini I. Chevance S. Del Conte R. Lalli D. Turano P. (2011). The anti-apoptotic Bcl-xL protein, a new piece in the puzzle of cytochrome c interactome. *PLoS One* 6:e18329. 10.1371/journal.pone.0018329 21533126 PMC3080137

[B4] Bo L. Cui H. Fang Z. Qun T. Xia C. (2016). Inactivation of transforming growth factor-β-activated kinase 1 promotes taxol efficacy in ovarian cancer cells. *Biomed. Pharmacother.* 84 917–924. 10.1016/j.biopha.2016.09.105 27764753

[B5] Chan K. K. L. Yao T. J. Jones B. Zhao J. F. Ma F. K. Leung C. Y. (2011). The use of Chinese herbal medicine to improve quality of life in women undergoing chemotherapy for ovarian cancer: a double-blind placebo-controlled randomized trial with immunological monitoring. *Ann. Oncol.* 22 2241–2249. 10.1093/annonc/mdq749 21355071

[B6] Chen M. May B. H. Zhou I. W. Sze D. M. Y. Xue C. C. Zhang A. L. (2016). Oxaliplatin-based chemotherapy combined with traditional medicines for neutropenia in colorectal cancer: a meta-analysis of the contributions of specific plants. *Crit. Rev. Oncol. Hematol.* 105 18–34. 10.1016/j.critrevonc.2016.07.002 27497028

[B7] Chen Y. Song M. Wang Y. Xiong W. Zeng L. Zhang S. (2015). The anti-DHAV activities of *Astragalus* polysaccharide and its sulfate compared with those of BSRPS and its sulfate. *Carbohydr. Polym.* 117 339–345. 10.1016/j.carbpol.2014.09.071 25498644

[B8] El-Khattouti A. Selimovic D. Haïkel Y. Megahed M. Gomez C. R. Hassan M. (2014). Identification and analysis of CD133+ melanoma stem-like cells conferring resistance to taxol: an insight into the mechanisms of their resistance and response. *Cancer Lett.* 343 123–133. 10.1016/j.canlet.2013.09.024 24080340

[B9] Han Q. B. (2018). Critical problems stalling progress in natural bioactive polysaccharide research and development. *J. Agric. Food Chem.* 66 4581–4583. 10.1021/acs.jafc.8b00493 29659260

[B10] Hong F. Yan J. Baran J. T. Allendorf D. J. Hansen R. D. Ostroff G. R. (2004). Mechanism by which orally administered β-1, 3-glucans enhance the tumoricidal activity of antitumor monoclonal antibodies in murine tumor models. *J. Immunol.* 173 797–806. 10.4049/jimmunol.173.2.797 15240666

[B11] Howard C. B. (2016). Chemo-preventive efficacy of a natural product anti-cancer agent which surpasses Taxol. *Cancer Res.* 76 2606–2606. 10.1158/1538-7445.am2016-2606

[B12] Jiao L. Dong C. Liu J. Chen Z. Zhang L. Xu J. (2017). Effects of Chinese medicine as adjunct medication for adjuvant chemotherapy treatments of non-small cell lung cancer patients. *Sci. Rep.* 7:46524. 10.1038/srep46524 28436479 PMC5402288

[B13] Jung Y. Jerng U. Lee S. (2016). A systematic review of anticancer effects of *Radix Astragali*. *Chin. J. Integr. Med.* 22 225–236. 10.1007/s11655-015-2324-x 26643507

[B14] Kampan N. C. Madondo M. T. McNally O. M. Quinn M. Plebanski M. (2015). Paclitaxel and its evolving role in the management of ovarian cancer. *Biomed. Res. Int.* 2015:413076. 10.1155/2015/413076 26137480 PMC4475536

[B15] Khorasanizadeh S. (2004). The nucleosome: from genomic organization to genomic regulation. *Cell* 116 259–272. 10.1016/S0092-8674(04)00044-3 14744436

[B16] Kober K. Mastick J. Paul S. Topp K. Smoot B. Abrams G. (2017). (431) characteristics of chemotherapy induced neuropathy (CIN) in cancer survivors who received taxol. *J. Pain* 18:S82 10.1016/j.jpain.2017.02.281

[B17] Lam W. Bussom S. Guan F. Jiang Z. Zhang W. Gullen E. A. (2010). The four-herb Chinese medicine PHY906 reduces chemotherapy-induced gastrointestinal toxicity. *Sci. Transl. Med.* 2:45ra59. 10.1126/scitranslmed.3001270 20720216

[B18] Lee Y. W. Chen T. L. Shih Y. R. V. Tsai C. L. Chang C. C. Liang H. H. (2014). Adjunctive traditional Chinese medicine therapy improves survival in patients with advanced breast cancer: a population-based study. *Cancer* 120 1338–1344. 10.1002/cncr.28579 24496917

[B19] Liu P. Zhao H. Luo Y. (2017). Anti-aging implications of *Astragalus Membranaceus* (Huangqi): a well-known chinese tonic. *Aging Dis.* 8 868–886. 10.14336/ad.2017.0816 29344421 PMC5758356

[B20] Liu S. H. Cheng Y. C. (2012). Old formula, new Rx: the journey of PHY906 as cancer adjuvant therapy. *J. Ethnopharmacol.* 140 614–623. 10.1016/j.jep.2012.01.047 22326673

[B21] Liu X. Xiu L. J. Jiao J. P. Zhao J. Zhao Y. Lu Y. (2017). Traditional Chinese medicine integrated with chemotherapy for stage IV non-surgical gastric cancer: a retrospective clinical analysis. *Chin. J. Integr. Med.* 15 469–475. 10.1016/S2095-4964(17)60377-7 29103417

[B22] Liu Z. Xiao Y. Ning S. Li Z. Y. Zhu Y. Hu G. (2016). Effect of taxol on the expression of FoxM1 ovarian cancer-associated gene. *Oncol. Lett.* 11 4035–4039. 10.3892/ol.2016.4490 27313736 PMC4888128

[B23] Magidson V. He J. Ault J. G. O’Connell C. B. Yang N. Tikhonenko I. (2016). Unattached kinetochores rather than intrakinetochore tension arrest mitosis in taxol-treated cells. *J. Cell Biol.* 212 307–319. 10.1083/jcb.201412139 26833787 PMC4748573

[B24] McCormick B. Lowes D. A. Colvin L. Torsney C. Galley H. F. (2016). MitoVitE, a mitochondria-targeted antioxidant, limits paclitaxel-induced oxidative stress and mitochondrial damage in vitro, and paclitaxel-induced mechanical hypersensitivity in a rat pain model. *Br. J. Anaesth.* 117 659–666. 10.1093/bja/aew309 27799181

[B25] Miller K. Wang M. Gralow J. Dickler M. Cobleigh M. Perez E. A. (2007). Paclitaxel plus bevacizumab versus paclitaxel alone for metastatic breast cancer. *N. Engl. J. Med.* 357 2666–2676. 10.1056/NEJMoa072113 18160686

[B26] Notte A. Rebucci M. Fransolet M. Roegiers E. Genin M. Tellier C. (2015). Taxol-induced unfolded protein response activation in breast cancer cells exposed to hypoxia: ATF4 activation regulates autophagy and inhibits apoptosis. *Int. J. Biochem. Cell Biol.* 62 1–14. 10.1016/j.biocel.2015.02.010 25724736

[B27] Park S. H. Seong M. A. Lee H. Y. (2016). p38 MAPK-induced MDM2 degradation confers paclitaxel resistance through p53-mediated regulation of EGFR in human lung cancer cells. *Oncotarget* 7 8184–8199. 10.18632/oncotarget.6945 26799187 PMC4884985

[B28] Pellegrini F. Budman D. R. (2005). Tubulin function, action of antitubulin drugs, and new drug development. *Cancer Invest.* 23 264–273. 10.1081/CNV-200055970 15948296

[B29] Peltier S. Oger J. M. Lagarce F. Couet W. Benoît J. P. (2006). Enhanced oral paclitaxel bioavailability after administration of paclitaxel-loaded lipid nanocapsules. *Pharm. Res.* 23 1243–1250. 10.1007/s11095-006-0022-2 16715372

[B30] Razis E. D. Fountzilas G. (2001). Paclitaxel: epirubicin in metastatic breast cancer—a review. *Ann. Oncol.* 12 593–598. 10.1023/a:1011108807105 11432615

[B31] Reichman B. S. Seidman A. D. Crown J. P. Heelan R. Hakes T. B. Lebwohl D. E. (1993). Paclitaxel and recombinant human granulocyte colony-stimulating factor as initial chemotherapy for metastatic breast cancer. *J. Clin. Oncol.* 11 1943–1951. 10.1200/jco.1993.11.10.1943 7691998

[B32] Ruttala H. B. Ko Y. T. (2015). Liposomal co-delivery of curcumin and albumin/paclitaxel nanoparticle for enhanced synergistic antitumor efficacy. *Colloids Surf. B Biointerfaces* 128 419–426. 10.1016/j.colsurfb.2015.02.040 25797481

[B33] Shi J. Zhou Y. Huang H. C. Mitchison T. J. (2011). Navitoclax (ABT-263) accelerates apoptosis during drug-induced mitotic arrest by antagonizing Bcl-xL. *Cancer Res.* 71 4518–4526. 10.1158/0008-5472.can-10-4336 21546570 PMC3129452

[B34] Sikov W. M. Berry D. A. Perou C. M. Singh B. Cirrincione C. T. Tolaney S. M. (2015). Impact of the addition of carboplatin and/or bevacizumab to neoadjuvant once-per-week paclitaxel followed by dose-dense doxorubicin and cyclophosphamide on pathologic complete response rates in stage II to III triple-negative breast cancer: CALGB 40603 (Alliance). *J. Clin. Oncol.* 33 13–21. 10.1200/jco.2014.57.0572 25092775 PMC4268249

[B35] Song J. Z. Yiu H. H. Qiao C. F. Han Q. B. Xu H. X. (2008). Chemical comparison and classification of Radix Astragali by determination of isoflavonoids and astragalosides. *J. Pharm. Biomed. Anal.* 47 399–406. 10.1016/j.jpba.2007.12.036 18272311

[B36] Visconti R. Grieco D. (2017). Fighting tubulin-targeting anticancer drug toxicity and resistance. *Endocr. Relat. Cancer* 24 T107–T117. 10.1530/ERC-17-0120 28808045

[B37] Wani M. C. Horwitz S. B. (2014). Nature as a remarkable chemist: a personal story of the discovery and development of Taxol. *Anticancer Drugs* 25 482–487. 10.1097/CAD.0000000000000063 24413390 PMC3980006

[B38] Watanabe A. Tanabe A. Maruoka R. Nakamura K. Hatta K. Ono Y. J. (2015). Fibrates protect against vascular endothelial dysfunction induced by paclitaxel and carboplatin chemotherapy for cancer patients: a pilot study. *Int. J. Clin. Oncol.* 20 829–838. 10.1007/s10147-014-0779-y 25539886

[B39] Weaver B. A. (2014). How taxol/paclitaxel kills cancer cells. *Mol. Biol. Cell* 25 2677–2681. 10.1091/mbc.E14-04-0916 25213191 PMC4161504

[B40] Wei W. Xiao H. T. Bao W. R. Ma D. L. Leung C. H. Han X. Q. (2016). TLR-4 may mediate signaling pathways of *Astragalus* polysaccharide RAP induced cytokine expression of RAW264. 7 cells. *J. Ethnopharmacol.* 179 243–252. 10.1016/j.jep.2015.12.060 26743224

[B41] Yang C. P. H. Horwitz S. B. (2017). Taxol: the first microtubule stabilizing agent. *Int. J. Mol. Sci.* 18:E1733. 10.3390/ijms18081733 28792473 PMC5578123

[B42] Yang C. P. H. Yap E. H. Xiao H. Fiser A. Horwitz S. B. (2016). 2-(m-Azidobenzoyl) taxol binds differentially to distinct β-tubulin isotypes. *Proc. Natl. Acad. Sci. U.S.A.* 113 11294–11299. 10.1073/pnas.1613286113 27651486 PMC5056068

[B43] Yin J. Y. Chan B. C. L. Yu H. Lau I. Y. K. Han X. Q. Cheng S. W. (2012). Separation, structure characterization, conformation and immunomodulating effect of a hyperbranched heteroglycan from Radix Astragali. *Carbohydr. Polym.* 87 667–675. 10.1016/j.carbpol.2011.08.045 34663019

[B44] Yoneyama H. Sasaki H. Sugawara M. Sasaki J. Atsuda K. (2017). P3-089Risk factors for neutropenia during gemcitabine and nanoparticle albumin-bound paclitaxel combination chemotherapy. *Ann. Oncol.* 28(Suppl. 9):mdx621.051. 10.1093/annonc/mdx621.051 20585851

[B45] Zeng Q. Y. Zeng L. J. Huang Y. Huang Y. Q. Zhu Q. F. Liao Z. H. (2014). 8-60hIPP5 (m)-induced G2/M cell cycle arrest involves activation of ATM/p53/p21 (cip1/waf1) pathways and delayed cyclin B1 nuclear translocation. *Asian Pac. J. Cancer Prev.* 15 4101–4107. 10.7314/APJCP.2014.15.9.4101 24935604

[B46] Zhang X. Wang Y. Hua Y. Duan J. Chen M. Wang L. (2018). Kinetic control over supramolecular hydrogelation and anticancer properties of taxol. *Chem. Commun.* 54 755–758. 10.1039/C7CC08041G 29308499

[B47] Zhu Y. Zhou Y. Shi J. (2014). Post-slippage multinucleation renders cytotoxic variation in anti-mitotic drugs that target the microtubules or mitotic spindle. *Cell cycle* 13 1756–1764. 10.4161/cc.28672 24694730 PMC4111722

