# Supplementary material for: Astragalus Polysaccharide RAP Selectively Attenuates Paclitaxel-Induced Cytotoxicity Toward RAW 264.7 Cells by Reversing Cell Cycle Arrest and Apoptosis
Source: Front Pharmacol. 2019 Feb 11;9:1580. doi: 10.3389/fphar.2018.01580 (PMC6378367; doi:10.3389/fphar.2018.01580)
Supplement: Supplementary file 1 [file Data_Sheet_1.docx]

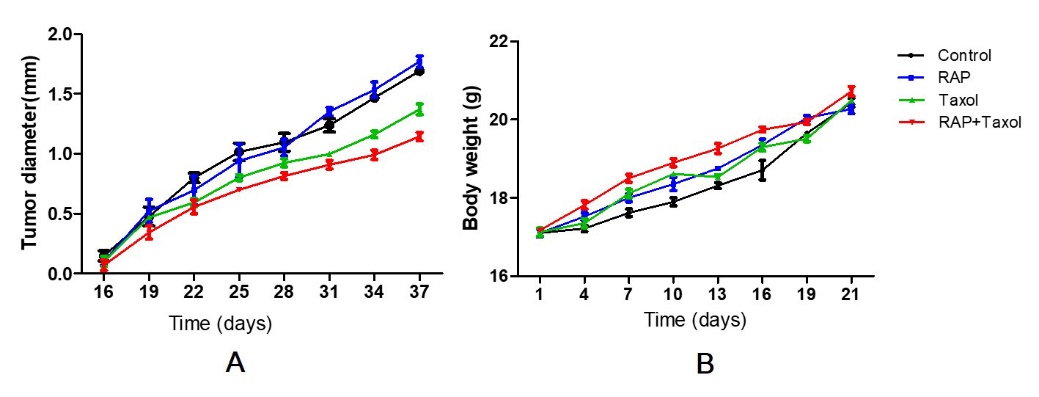


Fig S1. Tumor diameter (A) and body weight (B) of tumor-bearing mice treated with RAP with/without Taxol.


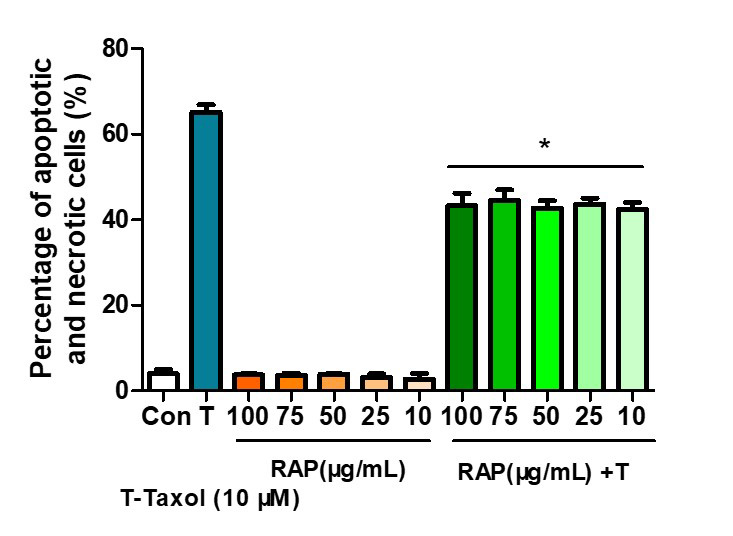


Fig S2. Apoptosis of RAW 264.7 cells treated by RAP (10-100 μg/mL) with/without Taxol (10 μM) detected by flow cytometry.

Data are presented as mean±SD. * p＜0.05 compared with Taxol alone group.
